# From Bench to Bedside: Translational Barriers in Mesenchymal Stem Cell Therapy for Intervertebral Disc Degeneration

**DOI:** 10.3390/bioengineering13050544

**Published:** 2026-05-09

**Authors:** Lidija Gradisnik, Borut Prestor, Uros Maver, Tomaz Velnar

**Affiliations:** 1 Institute of Biomedical Sciences, Medical Faculty of Maribor, 2000 Maribor, Slovenia; lidija.gradisnik@um.si (L.G.);; 2 Department of Neurosurgery, University Medical Centre Ljubljana, 1000 Ljubljana, Slovenia; borut.prestor@kclj.si; 3 Alma Mater Europaea University Maribor, 2000 Maribor, Slovenia

**Keywords:** mesenchymal stem cells, intervertebral disc, degenerative disc disease, regenerative medicine, translational research

## Abstract

Degenerative disc disease is a leading cause of chronic low back pain and disability worldwide, and current treatments primarily address symptoms rather than the underlying biological degeneration of the intervertebral disc. Mesenchymal stem cells (MSCs) have emerged as a promising regenerative approach due to their capacity for differentiation, immunomodulation, and secretion of bioactive factors that promote tissue repair. This review summarises findings from experimental and clinical studies investigating the therapeutic potential of MSC-based therapies for intervertebral disc regeneration, with particular focus on translational challenges that limit their clinical application. Preclinical studies generally show that MSC implantation can enhance extracellular matrix production, improve disc hydration, and modulate inflammatory processes within degenerated discs. Early clinical trials report improvements in pain and functional outcomes; however, consistent structural regeneration has not been reliably demonstrated. The limited clinical translation of MSC therapy is associated with several key challenges, including poor cell survival in the harsh disc microenvironment, variability in cell sources and manufacturing protocols, inadequate cell retention following intradiscal injection, and a lack of standardised outcome measures. In addition, regulatory and manufacturing barriers further complicate the development of reproducible and scalable MSC-based therapies. Although MSC-based therapies represent a promising strategy for the biological treatment of intervertebral disc degeneration, further research is required to improve cell survival, optimise delivery systems, standardise manufacturing procedures, and conduct large-scale controlled clinical trials to establish long-term safety and efficacy. Addressing these translational barriers will be essential for the successful integration of MSC-based therapies into clinical practice.

## 1. Introduction

Mesenchymal stem cell (MSC)-based therapies have emerged as a promising strategy in regenerative medicine, aiming to restore damaged tissues through differentiation, paracrine signalling, and modulation of the host microenvironment [1,2]. Increasing evidence suggests that the therapeutic effects of MSCs are mediated predominantly by the secretion of bioactive factors that promote tissue repair, regulate inflammation, and support the survival and function of resident cells, rather than by direct cellular replacement alone. The clinical success of haematopoietic stem cell transplantation in treating haematological disorders has demonstrated the broader therapeutic potential of stem-cell-based approaches and encouraged their investigation in other fields of medicine, including musculoskeletal and spinal disorders [3,4,5,6,7].

Among the various stem cell populations studied for regenerative applications, MSCs are of particular interest because of their accessibility, multipotent differentiation capacity, immunomodulatory properties, and relatively favourable safety profile [8,9,10]. MSCs can be isolated from several tissues, including bone marrow, adipose tissue, and umbilical cord, and are capable of differentiating into mesenchymal lineages such as osteocytes, chondrocytes, and adipocytes, making them especially relevant for musculoskeletal tissue repair [11,12,13,14,15,16,17]. In addition to their differentiation potential, MSCs secrete a broad range of cytokines, growth factors, and extracellular vesicles that influence the local tissue environment and support regeneration through paracrine mechanisms. Increasing evidence suggests that the therapeutic effects of MSCs are mediated predominantly through paracrine signalling rather than direct differentiation. The MSC secretome consists of a complex mixture of growth factors, cytokines, and extracellular vesicles that influence the local disc microenvironment and support tissue regeneration. Key bioactive factors include transforming growth factor-beta (TGF-β), which promotes extracellular matrix synthesis and supports discogenic differentiation, and growth differentiation factors such as GDF-5 and GDF-6, which are closely associated with the nucleus pulposus cell phenotype and cartilage-like matrix production. Insulin-like growth factor-1 (IGF-1) contributes to cell survival and proteoglycan synthesis, while anti-inflammatory cytokines such as interleukin-10 (IL-10) and tumour necrosis factor-stimulated gene-6 (TSG-6) help suppress inflammatory pathways that drive disc degeneration [16,17]. In addition, MSC-derived extracellular vesicles, including exosomes, play a crucial role in mediating intercellular communication by transferring proteins, lipids, and regulatory RNAs to resident disc cells. These vesicles can modulate gene expression, inhibit apoptosis, and enhance matrix production. While some factors, such as vascular endothelial growth factor (VEGF), may promote angiogenesis, their role in the largely avascular intervertebral disc remains complex and may be context-dependent. Overall, the coordinated action of these secretome components is thought to underlie the regenerative and immunomodulatory effects of MSC-based therapies in intervertebral disc degeneration [10,11,16,17].

Intervertebral disc degeneration is a major cause of chronic low back pain and disability worldwide and represents a substantial socioeconomic burden. Current treatment strategies, including pharmacological therapy, physical rehabilitation, and surgical intervention, primarily address symptoms rather than the underlying biological degeneration of the disc [18,19,20]. Consequently, increasing attention has been directed towards regenerative approaches that aim to restore disc structure and function. Among these, intradiscal MSC therapy has emerged as a potential strategy to slow degeneration, enhance extracellular matrix synthesis, modulate inflammation, and improve disc hydration and biomechanical function.

Despite encouraging results from experimental studies and early clinical trials, the clinical translation of MSC therapy for intervertebral disc degeneration remains limited [21,22]. The intervertebral disc is a particularly challenging environment for cell-based therapy because of its avascular nature, low oxygen tension, limited nutrient supply, acidic pH, and exposure to mechanical loading. These conditions reduce the survival and retention of transplanted cells and may significantly impair their regenerative capacity. In addition, substantial variability in cell source, expansion protocols, delivery techniques, and outcome measures complicates the comparison of published studies and hinders the development of standardised therapeutic strategies [3,7,23,24,25,26].

The aim of this review is to analyse the translational challenges associated with MSC therapy for intervertebral disc degeneration and to identify the main factors currently limiting its routine clinical application. Specifically, we examine the biological, biomechanical, and microenvironmental barriers that affect cell survival and regenerative potential, evaluate evidence from preclinical and clinical studies, and discuss current limitations related to cell manufacturing, delivery strategies, and regulatory standardisation. By integrating findings from experimental and clinical research, this review provides a focused overview of the key steps required to translate MSC-based therapy from bench to bedside in the treatment of degenerative disc disease.

## 2. Pathophysiology of Intervertebral Disc Degeneration

Intervertebral disc degeneration begins with a progressive decline in the population of large notochordal cells within the nucleus pulposus, resulting in altered cellular function and impaired disc homeostasis [27,28]. This initiates a degenerative cascade characterised by extracellular matrix breakdown, increased matrix metalloproteinase (MMP) activity, a shift from type II to type I collagen synthesis, and a reduction in proteoglycan content. As a consequence, the intervertebral disc progressively loses its ability to retain water, leading to disc dehydration, decreased turgor pressure, and structural collapse. These changes compromise the mechanical integrity of the spine, resulting in segmental instability and increased susceptibility to microtrauma [27,28,29,30,31].

As degeneration progresses, nucleus pulposus cells increase the production of pro-inflammatory and catabolic cytokines, including IL-1, IL-6, IL-12, IL-17, TNF-α, IFN-β, and IFN-γ, which further accelerate matrix degradation and cellular dysfunction [1,2,16,27,28,29,30]. These mediators contribute to angiogenesis, granulation tissue formation, fibrosis, and the activation of nociceptive nerve endings within the disc. Additional factors, such as ischaemia, mechanical stress, elevated catabolic enzyme activity, and reduced aggrecan content, further aggravate the degenerative process [2,16,30,31,32]. The accompanying proliferation of sensory nerve fibres may lead to abnormal disc innervation and the development of discogenic pain [2,16,30,31,32].

**Figure 1:**
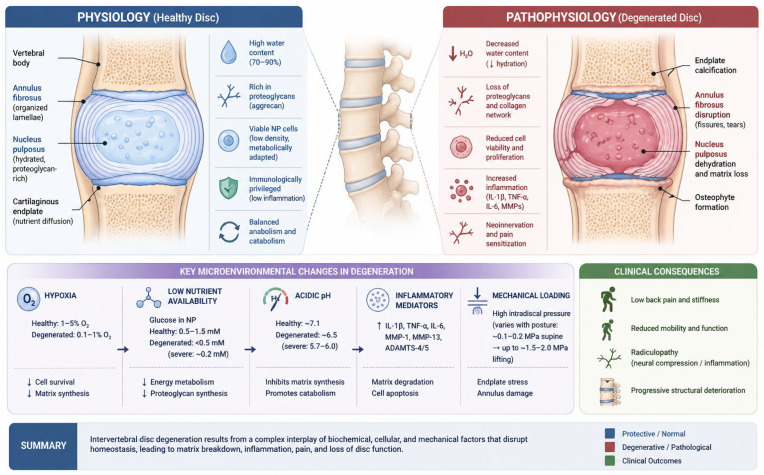
Intervertebral disc physiology and degeneration. The healthy disc is a hydrated, proteoglycan-rich structure that supports balanced cellular activity and matrix homeostasis. Degeneration is characterised by loss of hydration and matrix integrity, reduced cell viability, inflammation, and structural disruption. The degenerated microenvironment exhibits hypoxia, low nutrient availability, acidic pH, and increased mechanical loading, which impair cell function and promote matrix degradation. These changes ultimately lead to pain, reduced function, and progressive disc deterioration.

In later stages of degeneration, structural abnormalities such as disc protrusion, herniation, and nerve root compression may develop, contributing to worsening pain and neurological symptoms. Altered biomechanics and chronic mechanical overload may also promote osteophyte formation, further contributing to spinal canal narrowing and functional impairment [27,28,29,30]. The intervertebral disc physiology and pathophysiology are schematically summarised in Figure 1.

### 2.1. Clinical Burden and Limitations of Current Therapies

Clinically, low back pain is the most common manifestation of degenerative spine disease and remains one of the leading causes of disability worldwide. Conventional management strategies primarily focus on symptom control rather than reversing the underlying biological degeneration of the intervertebral disc. Conservative treatment typically includes pharmacological pain management, physical therapy, acupuncture, local nerve blocks, and lifestyle modifications [1,3].

When conservative measures fail to provide sufficient relief, surgical interventions such as microdiscectomy, artificial disc replacement, or spinal fusion may be considered. In severe cases, laminectomy, with or without spinal fusion, may be performed to decompress the spinal canal and relieve nerve compression [1,2,27]. Although these procedures may reduce pain and improve function in selected patients, they do not address the biological mechanisms driving disc degeneration. In addition, surgical treatment is associated with operative risks, postoperative complications, and variable long-term outcomes. Changes in spinal biomechanics following surgery may also accelerate degeneration of adjacent segments, potentially leading to recurrent symptoms and the need for further intervention [2,3,28].

### 2.2. Emerging Regenerative Approaches

Advances in the understanding of the molecular and cellular mechanisms underlying disc degeneration have stimulated growing interest in regenerative medicine. Unlike conventional therapies, regenerative strategies aim to restore the structural integrity and metabolic balance of the intervertebral disc by targeting key pathological processes, including inflammation, extracellular matrix degradation, and cellular loss [1,2]. Among these approaches, stem cell therapy has gained particular attention as a potential strategy for disc regeneration. Both experimental studies and clinical trials have investigated the intradiscal implantation of stem cells into degenerated intervertebral discs as a treatment for low back pain [2,27]. This approach aims not only to alleviate symptoms but also to promote tissue repair and restore disc function [2,27,28,29].

Stem cells are considered promising candidates for intervertebral disc regeneration because of their capacity for differentiation, tissue repair, and modulation of the local microenvironment [28,29,30]. In addition to differentiating into disc-like cells, they can secrete anti-inflammatory and trophic factors that support matrix synthesis and counteract degenerative changes. Preclinical studies and early clinical trials have reported encouraging findings, including improvements in pain, functional outcomes, and, in some cases, radiological indicators of disc regeneration [28,29,30,31].

Autologous stem cell transplantation, in which cells are harvested from the patient’s own tissue and injected intradiscally, offers the advantage of minimising the risk of immune rejection or disease transmission [29]. Furthermore, the minimally invasive nature of this procedure may reduce postoperative pain, shorten recovery time, and lower complication rates compared with conventional surgical treatment [29,30,31,32,33]. Although current evidence suggests that stem cell therapy is technically feasible and appears to be relatively safe, the long-term durability of its therapeutic effects remains uncertain. Nevertheless, by targeting the underlying biological mechanisms of degeneration rather than merely alleviating symptoms, stem-cell-based interventions may represent a promising disease-modifying strategy for degenerative disc disease. Continued research is required to determine their long-term efficacy and clinical applicability [30,31,32,33,34,35].

## 3. Intervertebral Disc Regeneration and Evidence from Animal Studies

Although cells of the nucleus pulposus constitute only approximately 1% of the total tissue volume of the intervertebral disc, they play a crucial role in maintaining the extracellular matrix (ECM), which is essential for disc structure, hydration, and biomechanical stability [27]. These cells regulate the synthesis and turnover of matrix components such as proteoglycans and type II collagen, enabling the disc to retain water and withstand compressive mechanical loads. Loss or dysfunction of nucleus pulposus cells during degeneration therefore represents a key therapeutic target for regenerative strategies [27,33].

One of the earliest attempts to restore disc cellularity was performed by Nishimura et al. in 1998 using a rat model [36]. In this pioneering study, cryopreserved autologous nucleus pulposus cells were reimplanted into degenerated discs, resulting in delayed degeneration of the annulus fibrosus and preservation of nucleus pulposus tissue compared with untreated controls [33,34]. This study provided early proof-of-concept that restoration of cellularity within the disc could slow degenerative progression. Subsequent animal and ex vivo studies, as well as preliminary human investigations, further suggested that autologous nucleus pulposus cell reimplantation may reduce degenerative changes, alleviate low back pain, and partially restore disc height and hydration compared with discectomy-only treatment [2,3].

Building on these early findings, regenerative strategies increasingly focused on the use of multipotent stem cells to replenish the degenerating disc microenvironment. Among the various stem cell types investigated, MSCs have emerged as the most promising candidates for intervertebral disc regeneration [5,6]. MSCs can be delivered percutaneously into the disc, where they may contribute to matrix restoration, modulation of inflammation, and regeneration of disc-like tissue. Under appropriate conditions, MSCs can acquire phenotypic characteristics similar to nucleus pulposus cells and contribute to extracellular matrix synthesis [10,16,17,18].

Both bone marrow-derived MSCs (BM-MSCs) and adipose-derived MSCs (AD-MSCs) have demonstrated the capacity to differentiate into nucleus pulposus-like cells in vitro. In addition to their differentiation potential, MSCs exert strong immunomodulatory and trophic effects through paracrine signalling. By secreting anabolic growth factors and cytokines, MSCs influence the behaviour of native nucleus pulposus cells, stimulate extracellular matrix synthesis, and suppress inflammatory pathways that contribute to disc degeneration [17,18,19,20,32]. Bone marrow remains the most widely used MSC source in experimental studies. In vitro, BM-MSCs are capable of differentiating into osteoblasts, adipocytes, and chondrocytes, and under appropriate stimulation conditions they can acquire phenotypic features resembling native nucleus pulposus cells [1,16,19,29]. Clarke et al. demonstrated that stimulation of BM-MSCs with growth differentiation factor-6 (GDF-6) significantly increased the expression of nucleus pulposus–specific genes, thereby promoting differentiation toward a discogenic phenotype [37].

Extensive in vivo animal experiments have provided further evidence supporting the regenerative potential of MSC therapy. Crevensten et al. reported radiographic enlargement of discs and increased cellularity following MSC implantation into degenerated rat intervertebral discs [38]. Similarly, Sakai et al. demonstrated in rabbit models that injected MSCs proliferated within the disc and differentiated into cells expressing markers characteristic of nucleus pulposus cells. These cells synthesised extracellular matrix components such as type II collagen and proteoglycans, leading to improved disc height and increased hydration as measured by imaging and histological analyses [39].

Beyond direct differentiation, MSCs also exert important paracrine and anti-inflammatory effects that contribute to the restoration of a more favourable regenerative microenvironment. Several studies have shown that MSCs secrete bioactive molecules capable of suppressing pro-inflammatory cytokines such as IL-6, IL-8, and TNF-α, while simultaneously promoting anabolic pathways involved in matrix synthesis [37,38,39]. These immunomodulatory effects may help counteract the chronic inflammatory environment characteristic of degenerated discs and support tissue repair [40,41,42].

Additional support for the therapeutic potential of MSC therapy has been demonstrated in large-animal models that more closely resemble human spinal biomechanics. For example, Steffen et al. investigated MSC injections in degenerated intervertebral discs of dogs [43]. Although significant radiological changes were not observed, treated animals exhibited measurable improvements in mobility and reduced pain behaviour. These findings suggest that MSC therapy may provide functional benefits even in the absence of clear structural regeneration, highlighting the importance of evaluating both functional and biological outcomes in regenerative studies.

More recent experimental studies have explored strategies to enhance the regenerative potential of MSC therapy. Approaches such as hypoxic preconditioning, genetic modification to promote discogenic differentiation, and the use of biomaterial scaffolds or hydrogels have been investigated to improve cell survival and retention within the disc [16,18,39]. These strategies aim to overcome the hostile microenvironment of the degenerated intervertebral disc, which is characterised by low oxygen levels, limited nutrient supply, acidic pH, and high mechanical loading. By providing structural support and biochemical cues, biomaterial-based delivery systems may further enhance the regenerative capacity of transplanted cells [39,40,41,42].

Collectively, in vitro and in vivo animal studies demonstrate the potential of MSC-based therapies to promote regeneration of degenerated intervertebral discs. Reported outcomes include improved extracellular matrix composition, increased disc height, improved hydration indicated by enhanced T2-weighted MRI signals, and suppression of inflammatory mediators. Findings from studies by Crevensten, Sakai, Teixeira, Miguélez-Rivera, and Steffen support the concept that MSCs may contribute to both structural and functional improvement in degenerated discs [40,41,42,43].

However, it is important to recognise that the majority of these investigations remain preclinical or rely on ex vivo experimental models. Differences in biomechanics, disc size, immune responses, and disease chronicity between animal models and human patients may significantly influence treatment outcomes. As a result, findings from animal studies cannot be directly extrapolated to clinical practice. These limitations highlight the importance of translational research aimed at bridging the gap between promising experimental results and consistent clinical outcomes in patients with degenerative disc disease [41]. A summary of representative preclinical animal studies investigating stem-cell-based regeneration of the intervertebral disc is presented in Table 1.

## 4. Human Trials for Intervertebral Disc Regeneration

Although preclinical studies have demonstrated encouraging regenerative effects of MSC therapy in experimental models of intervertebral disc degeneration, its clinical translation remains an area of active investigation. Over the past two decades, several early-phase clinical studies have explored the feasibility, safety, and therapeutic potential of MSC-based interventions for degenerative disc disease. While preliminary outcomes have generally been favourable, significant uncertainties remain regarding optimal cell sources, dosing strategies, delivery methods, and long-term therapeutic durability. Consequently, MSC therapy for degenerative disc disease has not yet been widely adopted in routine clinical practice [44,45,46].

One of the earliest clinical investigations was conducted by Yoshikawa et al., who treated two patients with autologous BM-MSCs [47]. Both patients experienced reductions in pain and improved T2-weighted MRI signals within the treated discs, suggesting early signs of improved disc hydration and potential regenerative activity. However, the extremely small sample size limits the generalisability of these findings, and the study primarily demonstrated feasibility rather than definitive therapeutic efficacy.

Subsequent clinical studies expanded on these preliminary observations. In a study by Orozco et al., ten patients with chronic discogenic low back pain received intradiscal injections of autologous MSCs [48]. Although MRI analysis did not demonstrate a statistically significant increase in disc height, improved disc hydration was observed, and approximately 85% of participants reported reductions in pain within the first three months following treatment. These improvements were accompanied by enhanced functional capacity and overall quality of life. Similarly, Pettine et al. evaluated autologous BM-MSC injections in a cohort of 26 patients and reported significant reductions in pain scores in most participants, along with improvements in disability indices and functional outcomes. An additional challenge is the variability in dosing for intradiscal MSC therapy, as a clearly defined minimum effective dose (MED) has not yet been established [49].

Additional observational studies have provided further evidence supporting the potential clinical benefits of MSC therapy. Centeno et al. reported improvements in both clinical symptoms and radiological findings in a cohort of 33 patients treated with autologous MSC injections [50]. Reductions in disc protrusion and improvements in disc morphology were observed on imaging, suggesting possible structural benefits in addition to symptomatic relief. Elabd et al. investigated the use of BM-MSCs cultured under hypoxic conditions prior to implantation [51]. Hypoxic preconditioning is believed to enhance MSC survival and functional activity within the low-oxygen environment of the intervertebral disc. In this study, five patients received intradiscal injections of hypoxia-preconditioned MSCs, and most participants reported clinical improvement, including increased mobility and reduced pain. These findings suggest that optimisation of cell preparation protocols may influence therapeutic outcomes.

Despite these encouraging findings, several methodological limitations constrain the interpretation of early clinical studies. Most investigations involved small patient cohorts and lacked well-defined control groups or randomisation. In addition, variability in patient selection criteria, MSC sources, culture conditions, and injection protocols complicates direct comparison between studies. These limitations make it difficult to determine whether reported improvements are attributable to stem cell therapy itself or to confounding factors such as placebo effects, natural fluctuations in symptoms, or concurrent conservative treatments [18,45,46,47,48,49,50,51,52].

More recent studies have attempted to address these limitations through improved study design. Noriega et al. conducted a controlled clinical trial comparing MSC-treated patients with a control group receiving conventional treatment [49,50]. The authors reported significant improvements in visual analogue scale (VAS) pain scores and MRI-based parameters in the MSC-treated group. Although these findings provided stronger evidence for a therapeutic effect, the relatively small sample size and heterogeneity among participants still limited the statistical power and generalisability of the results.

A larger randomised controlled trial was later conducted by Amirdelfan et al., involving 100 patients with chronic discogenic low back pain [53]. This study demonstrated statistically significant reductions in pain intensity and improvements in functional outcomes among patients receiving MSC therapy compared with control groups. The authors also reported a favourable safety profile, with no major adverse events associated with the procedure. Nevertheless, the study highlighted several remaining challenges, including variability in cell preparation and delivery techniques, potential selection bias, and the need for longer follow-up periods to evaluate the durability of therapeutic effects.

Another important limitation of current clinical research relates to outcome assessment. Many studies rely heavily on patient-reported outcomes, such as pain scores and functional questionnaires. While these measures are essential for evaluating clinical benefit, they are inherently subjective and may not directly reflect structural regeneration of the intervertebral disc. Objective assessments, including advanced imaging techniques such as quantitative MRI, T2 mapping, and biochemical biomarkers of disc metabolism, are therefore increasingly recognised as important complementary tools for evaluating structural and biological changes within the disc [49,50].

Taken together, the current body of clinical evidence suggests that MSC-based therapies may provide meaningful improvements in pain and functional outcomes in patients with degenerative disc disease. However, consistent structural regeneration has not yet been reliably demonstrated, and the mechanisms underlying clinical improvement remain incompletely understood. It remains unclear whether therapeutic effects are primarily related to structural regeneration, modulation of inflammation, or paracrine signalling mechanisms [42,43,44,45,46,49,50].

To establish MSC therapy as a reliable clinical treatment for degenerative disc disease, further large-scale, multicentre, randomised controlled trials will be required. Future studies should aim to standardise cell preparation methods, optimise dosing and delivery strategies, and incorporate objective imaging and molecular biomarkers to evaluate therapeutic responses. Long-term follow-up will also be essential to determine the durability of treatment effects and to fully assess the safety profile of MSC-based interventions. Only through rigorous and standardised clinical evaluation can the true therapeutic potential of MSC therapy for degenerative disc disease be determined [42,43,44,45,46]. A summary of the major clinical studies investigating MSC therapy for degenerative disc disease is presented in Table 2.

### 4.1. Comparison of Clinical Trials

Despite generally favourable outcomes reported across clinical studies, substantial heterogeneity exists among published trials, complicating direct comparison of results and limiting the development of standardised treatment protocols [47,48,49]. One major difference between studies is the source and preparation of mesenchymal stem cells, with some investigators using autologous bone marrow-derived MSCs, others using adipose-derived MSCs, and some employing culture-expanded cells while others use bone marrow concentrate. These variations may significantly influence cell viability, regenerative potential, and paracrine activity. In addition, the number of injected cells varies considerably between studies, ranging from approximately 10^6^ to over 10^7^ cells per disc, and the optimal therapeutic dose has not yet been clearly established [48,49,50]. Patient selection criteria also differ among studies, particularly regarding the stage of disc degeneration, patient age, and presence of Modic changes, all of which may influence treatment outcomes. Furthermore, outcome assessment methods are not standardised across clinical trials [43,44,45,46,47,48,49]. Many studies rely primarily on patient-reported pain and disability scores, whereas fewer include objective imaging techniques such as quantitative MRI or T2 mapping to evaluate structural disc regeneration. Differences in follow-up duration further complicate interpretation, as some studies report outcomes after only 6 to 12 months, while true disc regeneration may require several years to become detectable. Taken together, these methodological differences represent a major limitation of current clinical research and highlight the need for standardised clinical trial design, including consistent cell preparation protocols, dosing strategies, patient selection criteria, and objective outcome measures. The lack of standardisation across clinical studies currently represents one of the most significant barriers to the clinical translation of MSC therapy for intervertebral disc degeneration [47,48,49,50].

### 4.2. Minimum Effective Dose for Intradiscal Injection in Regenerative Medicine

Another source of heterogeneity across clinical studies is the variability in the number of mesenchymal stem cells administered per disc. Reported doses typically range from approximately 1 × 10^6^ to over 1 × 10^7^ cells per injection, although exact values are not consistently standardised across studies [49]. For example, studies using bone marrow concentrate often deliver lower effective cell numbers compared with culture-expanded MSC preparations, which may reach higher cell counts prior to implantation. Despite this variability, most studies report clinical improvements in pain and functional outcomes, suggesting that a broad therapeutic window may exist [47,48,49,50].

However, the minimum effective dose (MED) required to achieve consistent clinical benefit remains undefined. The absence of dose–response studies, combined with heterogeneity in cell sources, expansion protocols, and patient selection criteria, makes it difficult to determine whether higher cell numbers translate into improved therapeutic efficacy [46,47,48,49,50]. In addition, factors such as cell viability, retention within the disc, and paracrine activity may be more critical determinants of clinical outcome than absolute cell number alone. These considerations highlight the need for standardised dosing strategies and well-designed clinical trials specifically aimed at establishing dose–response relationships and identifying the optimal therapeutic dose for intradiscal MSC therapy [49,50,51].

## 5. Bridging the Gap Between Preclinical and Clinical Outcomes

**Figure 2:**
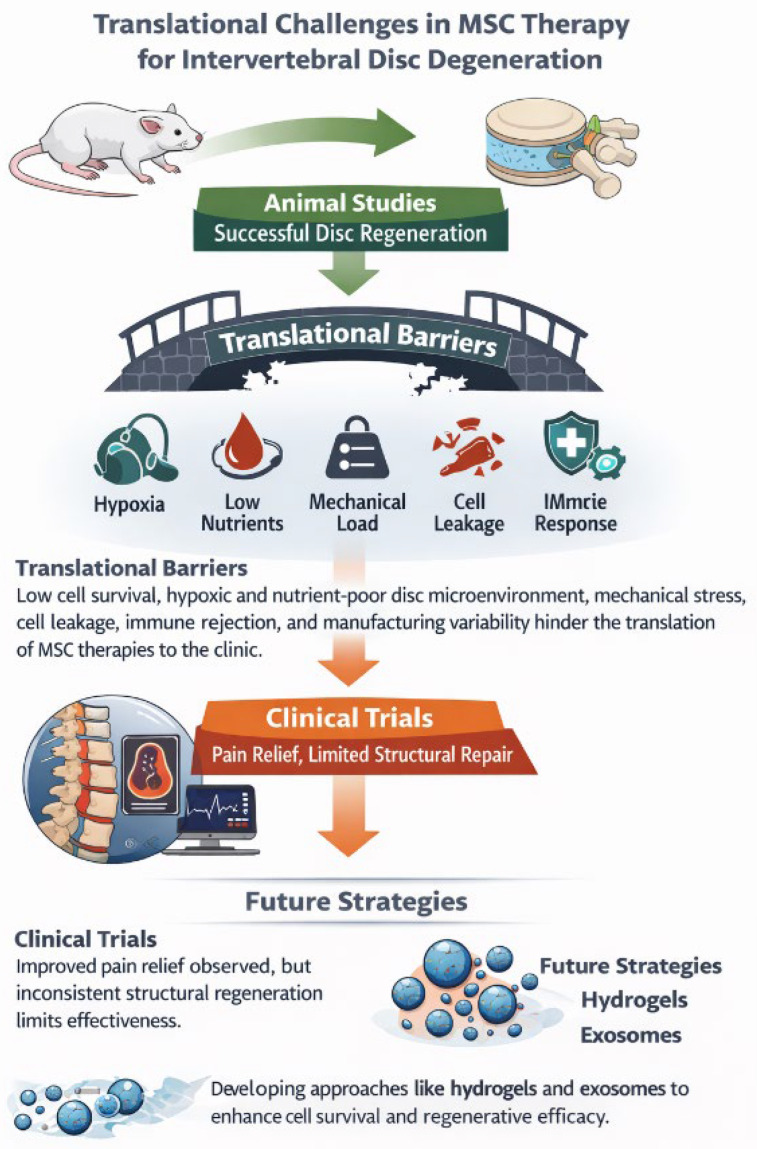
Translational pathway and major barriers in MSC therapy for intervertebral disc degeneration. Preclinical animal studies have demonstrated promising regenerative effects of MSC therapy, including increased extracellular matrix production, improved disc hydration, and partial restoration of disc structure. However, translation to clinical practice is limited by several barriers, including the harsh intervertebral disc microenvironment (hypoxia, low nutrient availability, acidic pH), high mechanical loading, cell leakage following intradiscal injection, immune and inflammatory responses, and variability in cell manufacturing and preparation protocols. As a result, clinical trials have shown consistent improvements in pain and functional outcomes but inconsistent structural regeneration of the intervertebral disc. Emerging strategies, including biomaterial-based delivery systems such as hydrogels and cell-free therapies based on MSC-derived exosomes, aim to overcome these translational barriers and improve clinical outcomes.

Despite the consistent success of MSC therapy in experimental animal models, translation into reliable clinical efficacy in humans has proven considerably more challenging. While preclinical studies frequently demonstrate improved disc structure, increased extracellular matrix synthesis, and restoration of disc hydration, these outcomes have not been reproduced with the same consistency in clinical trials. Several biological, biomechanical, and methodological factors contribute to this discrepancy [51,52]. Translational pathway and major barriers in MSC therapy for intervertebral disc degeneration are summarised in Figure 2. A brief comparison of the results of preclinical and clinical outcomes is summarised in Table 3.

A key aspect of understanding the therapeutic potential of MSCs is the mechanism by which they exert their regenerative effects. The mechanisms underlying MSC-mediated regeneration remain under investigation, with two primary hypotheses: direct differentiation and paracrine signalling [50,51]. Early studies suggested that MSCs contribute to tissue repair by differentiating into nucleus pulposus-like cells and directly replacing damaged tissue. However, increasing evidence indicates that the therapeutic effects of MSCs are predominantly mediated through paracrine mechanisms. The MSC secretome, including growth factors (e.g., TGF-β, GDF-5), cytokines, and extracellular vesicles, plays a critical role in modulating the local microenvironment by promoting extracellular matrix synthesis, inhibiting apoptosis, and suppressing inflammation. Rather than being mutually exclusive, these mechanisms may act in a complementary manner, with paracrine signalling dominating the early therapeutic response and differentiation contributing to longer-term tissue remodelling under favourable conditions. Importantly, the harsh microenvironment of the degenerated intervertebral disc may limit the extent of MSC differentiation in vivo, further supporting the concept that MSCs primarily function as “biological modulators” rather than direct structural replacements. This distinction is particularly relevant in the context of the intervertebral disc, where the harsh microenvironment may favour paracrine-mediated effects over direct cellular replacement [53,54].

One of the most important differences lies in the anatomical and biomechanical characteristics of the intervertebral disc across species. Commonly used animal models, including rodents and rabbits, possess relatively small and structurally simpler discs that experience substantially lower mechanical loads compared with the human spine. Human intervertebral discs are exposed to continuous axial compression, torsion, and shear forces generated during daily activities such as walking, bending, and lifting. These biomechanical stresses can negatively affect the survival and retention of transplanted cells. In contrast, implanted cells in small animal models are exposed to lower mechanical loads and more favourable diffusion gradients for oxygen and nutrients, which may enhance cell survival and integration into host tissue [53,54].

Another major challenge is the harsh biochemical microenvironment of the degenerated human intervertebral disc. The disc is the largest avascular structure in the human body and relies on passive diffusion through the cartilaginous endplates for nutrient supply. As degeneration progresses, the disc environment becomes increasingly hypoxic, acidic, and nutrient-deficient. Reduced glucose availability, accumulation of lactic acid, and decreased oxygen tension impair cellular metabolism and reduce the viability of implanted stem cells [5,6,55]. Although MSCs demonstrate some adaptation to hypoxic environments, the severe metabolic constraints present in advanced degenerative discs may significantly limit their regenerative capacity. Animal models often fail to fully replicate these metabolic conditions, which may partially explain the discrepancy between preclinical success and clinical outcomes [56]. The term harsh microenvironment should therefore be understood in quantitative terms. The healthy intervertebral disc already functions under low oxygen conditions, with oxygen tension in the nucleus pulposus often reported at approximately 1% to 5%, and this may decrease further with degeneration and endplate calcification [57]. Nutrient availability is similarly restricted because glucose and oxygen must diffuse through the cartilaginous endplates; glucose concentrations below approximately 0.5 mM have been associated with reduced disc cell viability, while further reductions toward 0.2 mM may be incompatible with long-term cell survival [58]. Degeneration is also associated with accumulation of lactic acid and progressive acidification, with pH values decreasing from approximately 7.1 in healthy discs to around 6.5, and in severe degeneration even lower values have been reported. These acidic conditions can inhibit matrix synthesis, reduce proteoglycan production, impair MSC proliferation, and alter differentiation capacity [58,59].

Biomechanical conditions add another layer of stress. Human lumbar discs are exposed to substantial intradiscal pressure during daily activities, with pressure increasing during sitting, flexion, lifting, and axial compression. Unlike small animal models, the human disc is subjected to repeated compression, torsion, and shear forces, which can reduce MSC retention, promote leakage through annular fissures or needle tracks, and impair cell survival. Therefore, the hostile disc microenvironment is not merely descriptive but reflects a combination of measurable metabolic and biomechanical constraints, including low oxygen tension, glucose deprivation, acidic pH, impaired diffusion, and high mechanical loading. These factors collectively reduce the probability that injected MSCs will survive long enough to exert sustained regenerative or paracrine effects [60].

Immunological and inflammatory responses also differ between experimental models and human patients. Laboratory animals often display reduced immune reactivity or are genetically modified to minimise immune rejection, and immunocompromised models are frequently used to improve stem cell engraftment and survival. In contrast, the human immune system may mount stronger or more prolonged inflammatory responses following cell implantation. Persistent inflammation within the degenerating disc can compromise stem cell survival, disrupt differentiation pathways, and alter the local microenvironment in ways that reduce therapeutic efficacy [61].

The complexity and chronicity of human degenerative disc disease further complicate clinical translation. In animal models, degeneration is typically induced through controlled mechanical injury, enzymatic degradation, or needle puncture of the disc. These acute injury models may respond more readily to regenerative interventions because the surrounding tissue retains a relatively intact biological environment. In contrast, human degenerative disc disease develops gradually over many years and is often associated with ageing, metabolic disorders, altered spinal biomechanics, and systemic inflammatory conditions [3,16,56,61].

An additional factor influencing the success of MSC-based therapy under mechanical loading conditions is the source of the transplanted cells. BM-MSCs are the most extensively studied and demonstrate strong chondrogenic and discogenic differentiation potential; however, their viability and matrix production may be significantly reduced under excessive mechanical stress and high intradiscal pressures [56,61,62]. AD-MSCs exhibit higher proliferative capacity and greater resistance to mechanical strain in vitro, but some studies suggest they may display comparatively lower expression of nucleus pulposus–specific markers under discogenic conditions. Umbilical cord-derived MSCs (UC-MSCs) have attracted increasing attention due to their enhanced proliferative potential, lower immunogenicity, and greater tolerance to hypoxic and mechanically challenging environments, which may improve their survival within the intervertebral disc [61,62].

Experimental studies indicate that moderate dynamic compression can promote extracellular matrix synthesis and chondrogenic differentiation across MSC types, whereas excessive mechanical loading reduces cell viability, induces apoptosis, and impairs regenerative capacity. Importantly, the threshold at which mechanical loading shifts from beneficial to detrimental may vary depending on the MSC source, with UC-MSCs potentially demonstrating greater resilience to mechanical and metabolic stress compared with adult tissue-derived MSCs. These differences highlight the importance of considering cell source selection in combination with biomechanical conditions when designing MSC-based therapies for intervertebral disc regeneration [53,54,61].

Another critical factor involves the retention and distribution of transplanted cells within the disc. The pressurised nature of the nucleus pulposus and the structural disruption present in degenerated discs can result in rapid leakage of injected cells through annular fissures or needle tracts. Several studies have suggested that a substantial proportion of implanted cells may be lost shortly after injection, thereby reducing the effective therapeutic dose [52].

Variability in stem cell preparation and delivery protocols also contributes to inconsistent clinical outcomes. Preclinical studies often use carefully controlled cell populations with defined culture conditions, whereas clinical applications may involve substantial heterogeneity in cell sources, expansion methods, and dosing strategies. Differences in donor age, MSC passage number, cryopreservation techniques, and injection protocols can influence cell viability, differentiation potential, and paracrine signalling activity [2,53,54].

Differences in study design and outcome measures represent another important translational barrier. Preclinical investigations commonly rely on histological analysis, biochemical markers of matrix synthesis, or imaging-based indicators such as disc height restoration and increased proteoglycan content. In contrast, clinical trials typically prioritise patient-reported outcomes, including pain intensity, disability scores, and quality of life assessments. Although these measures are clinically meaningful, they may not directly correlate with structural regeneration of disc tissue [2,61].

Addressing these translational challenges will require the development of more physiologically relevant experimental models and improved therapeutic strategies. Small animal models, particularly rat tail models, have been widely used in early preclinical studies of intervertebral disc degeneration due to their accessibility, low cost, and ease of experimental manipulation [16,17]. However, these models are increasingly recognised as having limited translational relevance and may present a potential pitfall when extrapolating results to human clinical applications. One major limitation is the substantially lower mechanical loading experienced by rat caudal discs compared with human lumbar discs. Rat tails are non-weight-bearing structures, and the discs are exposed to minimal axial compression, creating a biomechanical environment fundamentally different from the highly loaded human spine. As a result, transplanted MSCs may exhibit improved survival and regenerative activity in these models compared with the mechanically demanding conditions present in patients [17,20,56]. In addition to biomechanical differences, rat intervertebral discs are smaller and have shorter diffusion distances for oxygen and nutrients, which may facilitate cell survival and metabolic activity. Furthermore, many small animal models retain notochordal cells into adulthood, in contrast to humans, where these cells are largely lost early in life. Notochordal cells are known to exert protective and regenerative effects within the disc microenvironment, which may further enhance the apparent efficacy of regenerative therapies in animal models. Another important limitation is that degeneration in rat models is typically induced through acute injury, such as needle puncture, rather than representing the chronic, multifactorial degeneration observed in human patients. These acute models may respond more favourably to regenerative interventions due to the relatively preserved surrounding tissue environment and shorter disease duration [56,61].

In contrast, large animal models, including sheep, goats, and pigs, are increasingly favoured for translational research because they more closely replicate human spinal anatomy, disc size, and biomechanical loading conditions [17,62]. These models experience weight-bearing axial compression and more physiologically relevant mechanical stresses, which are critical factors influencing MSC survival, retention, and regenerative potential. Large animal models offer spinal anatomy and biomechanics more comparable to those of humans and may therefore provide more accurate predictions of clinical efficacy [17,20,62]. Moreover, large animal models allow for the evaluation of surgical delivery techniques, implant stability, and long-term functional outcomes under conditions that more closely mimic clinical reality. Consequently, while small animal models remain valuable for initial mechanistic studies, reliance on these systems alone may lead to an overestimation of therapeutic efficacy. Incorporation of large animal models is therefore essential for bridging the gap between promising preclinical findings and successful clinical translation [16,20,56,61,62]. In addition, biomechanically loaded ex vivo disc culture systems and advanced bioreactor models are increasingly being used to simulate physiological loading conditions while allowing for precise control of environmental parameters [62].

Future translational research should also focus on improving the survival and retention of transplanted cells within the disc. Strategies such as biomaterial scaffolds, hydrogel carriers, and controlled-release delivery systems may help stabilise implanted cells and protect them from the hostile disc environment. Preconditioning of MSCs under hypoxic conditions, genetic modification to enhance discogenic differentiation, and the use of extracellular vesicles or exosome-based therapies are emerging approaches that may improve regenerative outcomes [2,52,53,54].

**Figure 3:**
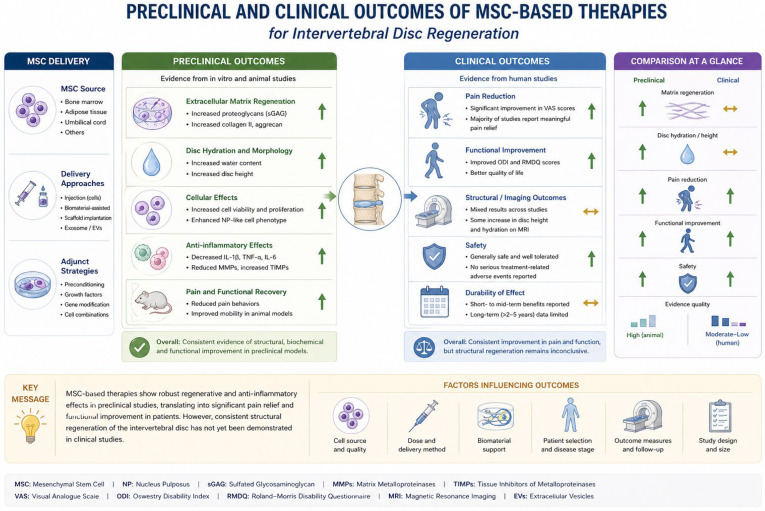
Preclinical and clinical outcomes of MSC-based therapies for intervertebral disc regeneration. Preclinical studies demonstrate consistent improvements in extracellular matrix synthesis, disc hydration, cell viability, and inflammation, resulting in functional recovery. In contrast, clinical studies show reliable pain reduction and functional improvement but inconsistent structural regeneration and limited long-term durability. Outcome variability is influenced by factors such as cell source, delivery method, biomaterial support, patient selection, and study design, highlighting a persistent translational gap between experimental and clinical findings.

Ultimately, bridging the gap between promising laboratory results and consistent clinical success will require an integrated approach combining advances in stem cell biology, biomaterial engineering, biomechanics, and clinical trial design. By developing more predictive preclinical models, standardising cell preparation protocols, and implementing rigorous translational research frameworks, the field can move closer to realising the full therapeutic potential of MSC-based therapies for degenerative disc disease [52,53,54,55,56]. The translational pathway from preclinical studies to clinical application is associated with multiple biological, biomechanical, and regulatory barriers that limit the clinical efficacy of MSC therapy (Table 4). Preclinical and clinical outcomes of MSC-based therapies for intervertebral disc regeneration are summarised in Figure 3.

## 6. Current Challenges

Despite the growing body of evidence supporting the potential of MSC therapy for intervertebral disc regeneration, numerous biological, technical, and translational challenges remain. These challenges must be addressed to achieve consistent and durable clinical outcomes and to bridge the gap between promising experimental findings and routine clinical application [1,6]. To move beyond descriptive reporting, the challenges associated with MSC-based therapy for intervertebral disc degeneration can be conceptualised within a problem-solution framework. Key barriers, including hypoxia, limited nutrient availability, mechanical loading, and cell leakage following intradiscal injection, directly impact MSC survival, retention, and function. These challenges can be systematically addressed through targeted bioengineering strategies: hypoxia and nutrient limitations may be mitigated through preconditioning or oxygen-responsive biomaterials; mechanical loading can be addressed using supportive scaffolds or bioreactor-based conditioning; and cell leakage may be reduced through injectable hydrogels or in situ forming biomaterials. This structured approach highlights that successful clinical translation depends not on a single intervention, but on the integration of multiple strategies tailored to the hostile disc microenvironment [1,2,3].

One of the key unresolved questions concerns the fate and functionality of MSCs after implantation. It remains unclear how long transplanted MSCs survive within the harsh disc microenvironment and whether they truly differentiate into discogenic cells or act primarily through paracrine mechanisms. The intervertebral disc is the largest avascular structure in the human body and is characterised by a hypoxic, acidic, and nutrient-deficient environment that worsens with age and endplate calcification. Under these conditions, transplanted cells face significant survival challenges [2]. In addition, mechanical loading, inflammation, and matrix degradation further contribute to the unfavourable microenvironment, hindering both cell survival and integration [2,4].

The longevity and behaviour of implanted MSCs have been investigated in several in vivo studies. Hang et al., using MRI and PET imaging in a canine model, reported an average MSC survival time of approximately three weeks following intradiscal injection [43]. The exact mechanism of regeneration also remains debated. While some evidence suggests that MSCs can differentiate into nucleus pulposus-like cells, other studies indicate that their primary therapeutic effects are mediated through paracrine signalling rather than direct cellular integration. Recent findings by Wang et al. support this paracrine hypothesis [63]. In rat models, hypoxic preconditioning of MSCs significantly improved cell survival, enhanced disc height, and increased type II collagen and aggrecan production while reducing apoptosis. These findings highlight the importance of optimising preconditioning protocols to improve MSC resilience and regenerative capacity. By exposing cells to environmental stimuli such as hypoxia, nutrient fluctuations, or growth factors prior to transplantation, it may be possible to enhance their adaptation to the avascular disc environment [48,63,64,65].

Another challenge involves the limited understanding of the differentiation pathways of human nucleus pulposus cells, which complicates the development of lineage-specific differentiation protocols. Advances in single-cell transcriptomics and proteomics have begun to identify molecular markers that may guide MSC differentiation towards a discogenic phenotype [34,66]. However, many recent studies have challenged the traditional assumption that implanted MSCs must replace damaged cells to achieve regeneration. Instead, increasing evidence suggests that MSCs exert their therapeutic effects primarily through paracrine signalling, releasing bioactive molecules such as cytokines, growth factors, and extracellular vesicles that influence native cell behaviour and tissue homeostasis [34,35,36,65,66].

Among these secreted factors, exosomes have gained particular attention. These nanosized extracellular vesicles facilitate intercellular communication by transferring functional molecules such as RNA, proteins, and lipids from MSCs to recipient cells [23,24,25,67]. Through this mechanism, exosomes can modulate gene expression, proliferation, apoptosis, and metabolic activity in recipient cells. As a result, exosome-based therapies represent a promising cell-free therapeutic approach that may overcome several limitations associated with direct MSC implantation, including immune rejection, limited cell survival, and variability in differentiation outcomes. Such approaches may enable the development of standardised, off-the-shelf biological products with improved safety and reproducibility [27,28,67].

Another major challenge involves the delivery and retention of MSCs within the intervertebral disc. Traditional intradiscal injection of cell suspensions often results in rapid cell loss due to leakage through annular fissures or needle tracts and poor retention within the pressurised disc environment [23,24,25]. To overcome these limitations, researchers are increasingly investigating biomaterial-based delivery systems, including hydrogels, microspheres, and injectable scaffolds that provide mechanical support and biochemical cues while protecting cells from hostile environmental conditions. These systems can also be engineered to release trophic factors or exosomes in a controlled manner, thereby enhancing regenerative signalling over time. The integration of tissue engineering approaches with MSC therapy holds significant promise for reconstructing disc structure and restoring biomechanical function [67,68,69,70,71,72,73].

In addition to biological and technical challenges, immunological and regulatory factors remain important barriers to clinical translation. Although MSCs possess immunomodulatory properties, their interaction with the disc’s immune-privileged environment is not yet fully understood. Autologous MSC therapy reduces the risk of immune rejection but is limited by donor age, variability, and processing time, whereas allogeneic MSCs offer greater scalability but may present safety and compatibility concerns. Furthermore, regulatory agencies classify MSC-based products differently across regions, complicating clinical approval and standardisation. Standardisation of cell sourcing, manufacturing, and quality control procedures is therefore essential to ensure reproducibility and clinical efficacy [50,51,52,53,67,68,69,70,71,72].

An important translational consideration in MSC-based therapy for intervertebral disc degeneration is the trade-off between minimally invasive delivery and optimal cell retention [2,6,28,67]. Intradiscal injection of MSCs is attractive due to its minimally invasive nature, reduced procedural risk, and potential for outpatient application under local anaesthesia. This approach aligns with current clinical practice, where minimally invasive interventions are preferred to limit tissue disruption and reduce recovery time. However, intradiscal injection has several important limitations, including poor cell retention within the nucleus pulposus, leakage through annular fissures or needle tracts, and reduced survival within the pressurised and avascular disc environment. In addition, the injection process itself may further disrupt annulus fibrosus integrity, potentially exacerbating cell loss and limiting the effective therapeutic dose. In contrast, biomaterial-supported strategies, including hydrogels, scaffolds, and tissue-engineered constructs, are designed to address these limitations by providing structural support and a protective microenvironment for transplanted cells [50,51,52,53,54]. These systems can improve cell retention by physically stabilising MSCs within the disc space, reduce leakage, and enhance cell survival by buffering against hypoxia, acidity, and mechanical stress. Furthermore, biomaterials can be engineered to provide biochemical cues that promote discogenic differentiation and enable sustained release of trophic factors or extracellular vesicles, thereby enhancing regenerative signalling over time [70,71,72]. Despite these advantages, scaffold-based approaches often require more invasive delivery methods, including open or minimally invasive surgical implantation, which may increase procedural complexity, operative risk, and recovery time. This introduces a clinical paradox, as the use of more complex implantation techniques may undermine one of the principal advantages of MSC therapy—its potential as a minimally invasive regenerative treatment [50,67,68,69,70,71].

This trade-off represents a fundamental challenge in the clinical translation of MSC-based therapies. From a clinical perspective, the optimal strategy must balance biological efficacy with procedural safety and feasibility. Injectable approaches prioritise simplicity, lower risk, and broader clinical applicability but may suffer from reduced therapeutic potency due to cell loss [26,42,67]. In contrast, scaffold-based strategies may achieve superior biological outcomes but face barriers related to invasiveness, cost, and surgical burden. Consequently, the choice of delivery method may ultimately depend on patient-specific factors, including the stage of disc degeneration, structural integrity of the annulus fibrosus, and overall surgical risk. Future developments are increasingly focused on hybrid solutions that aim to reconcile these competing priorities. Injectable biomaterials that undergo in situ gelation or solidification represent a promising approach, as they can be delivered through minimally invasive techniques while improving cell retention and mechanical stability. Similarly, advanced hydrogel systems, microsphere carriers, and bioresponsive materials capable of controlled release of cells or exosomes may offer a means to combine the advantages of both strategies. The development of such approaches will be critical for overcoming current translational barriers and achieving clinically effective and scalable MSC-based therapies for intervertebral disc regeneration [26,27,28,42,68,69,70,71,72]. Finally, long-term clinical follow-up data remain limited. While early-phase clinical trials have demonstrated improvements in pain and functional outcomes, radiological evidence of true disc regeneration remains inconsistent. Large-scale, randomised controlled trials with standardised outcome measures are required to evaluate long-term efficacy, durability of therapeutic effects, and safety. In summary, MSC therapy for intervertebral disc regeneration remains at a critical stage of development, and successful clinical translation will require coordinated advances in cell biology, biomaterial science, immunology, and biomechanics [26,27,42,67]. By optimising preconditioning protocols, improving delivery systems, harnessing exosome-based therapies, and addressing regulatory and clinical challenges, the field may move closer to achieving reliable regenerative treatment for degenerative disc disease [70,71,72,73].

### MSC-Based Therapy in the Context of Other Cell-Based Approaches

While MSCs are the most widely studied cell type for intervertebral disc regeneration, other cell sources have also been investigated and should be considered in a broader therapeutic context [27,42,70]. Native nucleus pulposus cells are intrinsically adapted to the disc microenvironment and can produce appropriate extracellular matrix components; however, their clinical use is limited by restricted availability and loss of phenotype during in vitro expansion. Chondrocyte-like cells have also been explored due to their matrix-producing capacity, but their ability to fully recapitulate the nucleus pulposus phenotype remains uncertain [50,51,52,70].

More recently, induced pluripotent stem cell (iPSC)-derived NP-like cells have gained attention due to their high differentiation potential and capacity to generate patient-specific cell populations. Nevertheless, concerns regarding tumourigenicity, differentiation control, and regulatory complexity currently limit their clinical translation. In comparison, MSCs offer several practical and biological advantages, including ease of isolation from multiple tissue sources, immunomodulatory properties, and the ability to exert regenerative effects through paracrine signalling. Rather than acting solely through direct differentiation, MSCs primarily function as bioactive modulators of the disc microenvironment, influencing resident cells and promoting tissue repair [50,51,52,53,67,68].

Taken together, these observations suggest that while alternative cell types may offer advantages in specific contexts, MSCs remain the most clinically relevant and translationally feasible option for intervertebral disc regeneration. Future strategies may also involve combinatorial approaches, integrating MSCs with NP cells or biomaterials to better mimic the native disc environment and enhance regenerative outcomes [70,71].

## 7. Standardisation and Regulatory Barriers

Beyond biological challenges, manufacturing and regulatory standardisation remain major bottlenecks in the clinical translation of MSC therapy. Ensuring the safety, reproducibility, and scalability of cell-based products requires strict adherence to Good Manufacturing Practice (GMP) standards, validated release criteria, and robust quality systems [74,75]. However, achieving such uniformity across research and clinical centres remains difficult, as many published studies still rely on heterogeneous laboratory-scale expansion protocols rather than harmonised clinical-grade manufacturing pipelines. Successful translation from bench to bedside therefore requires not only biological optimisation, but also standardised production, characterisation, and delivery procedures that meet increasingly stringent regulatory requirements [75,76,77].

A major source of inconsistency arises from variability in MSC source material and processing methods. MSCs can be isolated from bone marrow, adipose tissue, umbilical cord, dental pulp, synovium, and placental tissues, and these sources differ in proliferation kinetics, differentiation potential, immunomodulatory activity, and secretome composition [76,78]. Donor-related variables, including age and biological background, further influence cell fitness and function. In addition, differences in isolation procedures, serum supplementation, use of human platelet lysate versus foetal bovine serum, oxygen tension, passage number, and expansion conditions can substantially alter MSC phenotype and potency [3,79,80]. Even cryopreservation, thawing, and post-thaw handling may affect cell viability and function, thereby introducing additional batch-to-batch heterogeneity and complicating comparisons across studies [3,79,80,81,82,83,84].

The lack of standardised delivery and administration protocols also hinders translation. Intradiscal MSC therapy varies widely with respect to cell concentration, injected volume, needle gauge, injection rate, and the use of carriers or supportive biomaterials. These procedural differences are likely to influence cell retention, leakage, mechanical disruption of the annulus fibrosus, and ultimately therapeutic outcome. Without harmonised delivery parameters, it remains difficult to identify optimal dosing strategies or to compare efficacy across preclinical and clinical studies in a meaningful way [75,76].

From a regulatory perspective, MSC-based products occupy a complex space between biologics, tissue-engineered products, and advanced therapy medicinal products, depending on the level of manipulation and intended clinical use. Regulatory classification differs among jurisdictions, which complicates global development and multinational trials [4]. Comparative analyses have shown that the United States, European Union, and Japan apply distinct approval pathways and regulatory thresholds to stem cell therapies, with differences in definitions of minimal manipulation, homologous use, and post-marketing evidence requirements. While these frameworks are intended to balance innovation with safety, the lack of harmonisation increases development costs and slows clinical translation [77,83].

Quality control and batch consistency under GMP conditions add another layer of complexity. Although the International Society for Cell and Gene Therapy (ISCT) minimal criteria provide a foundational phenotypic definition of MSCs, these markers alone are insufficient to predict therapeutic efficacy. In practice, manufacturing centres must verify sterility, viability, identity, purity, and, ideally, potency for each production lot [83,84,85,86,87]. However, potency remains one of the most difficult attributes to standardise because MSCs act through multiple context-dependent mechanisms, including immunomodulation, trophic support, and paracrine signalling. Current potency assessment approaches, such as T-cell suppression assays, cytokine profiling, or gene-expression-based surrogate markers, are useful but labour-intensive and not yet universally validated across indications or manufacturing platforms [84,85,86].

Long-term safety surveillance is equally important. Although MSC-based products have generally demonstrated favourable short-term safety profiles, concerns remain regarding ectopic tissue formation, immune sensitisation, fibrotic effects, and possible tumour-supportive activity in permissive microenvironments. These considerations highlight the need for structured pharmacovigilance, long-term follow-up registries, standardised adverse event reporting, and traceability systems linking donor material, manufacturing batches, and treated patients [83,88].

Cost and scalability represent further translational barriers. GMP-compliant MSC production requires specialised infrastructure, trained personnel, validated clean-room facilities, and extensive documentation, all of which increase manufacturing costs. Emerging strategies such as automated closed-system bioreactors, xeno-free culture media, and GMP-compliant large-scale expansion protocols are expected to improve scalability and consistency, but these technologies are not yet uniformly adopted [76,89].

International collaboration will be essential to overcome these barriers. Consensus efforts led by organisations such as the International Society for Cell and Gene Therapy (ISCT) have helped define MSC identity and stimulate discussion around potency and release testing, but broader harmonisation is still needed. Shared reference materials, standardised manufacturing benchmarks, and collaborative multicentre trial frameworks could substantially improve reproducibility and reduce duplication of effort across institutions [85,86].

In parallel, ex vivo intervertebral disc organ culture systems are emerging as valuable translational platforms for evaluating standardised MSC-based therapies under physiologically relevant conditions. These models allow for controlled manipulation of oxygen tension, nutrient supply, pH, and mechanical loading, thereby improving the predictive value of preclinical testing [90,91]. Recent advances in biomechanically active bioreactors, organ culture systems, and physiologic loading platforms may help bridge the gap between in vitro optimisation and in vivo performance, particularly when combined with biomaterials, microfluidic technologies, and 3D biofabrication approaches [90,91,92].

Ultimately, advancing MSC therapy for intervertebral disc regeneration will require rigorous multicentre clinical trials designed around standardised manufacturing, delivery, and outcome assessment protocols. Integrated registries combining clinical, radiographic, and biomarker data will be important for evaluating long-term efficacy and safety [75,76,77]. Given the growing socioeconomic burden of degenerative disc disease and low back pain, addressing these scientific, manufacturing, and regulatory barriers through harmonisation, transparency, and international collaboration remains a critical priority [75,76].

### Regulatory Classification and International Frameworks for MSC-Based Therapies

Regulatory classification of MSC-based therapies varies significantly across major global regions, further complicating clinical translation, international standardisation, and the design of multicentre clinical trials. In the European Union, MSC-based products are generally classified as advanced therapy medicinal products (ATMPs) when they are substantially manipulated or intended for non-homologous use. As ATMPs, these products are regulated under the European Medicines Agency (EMA) framework and require centralised marketing authorisation, strict Good Manufacturing Practice (GMP) compliance, validated quality-control procedures, and evidence of safety, quality, and efficacy before clinical implementation [93,94].

In the United States, MSC-based therapies are regulated by the Food and Drug Administration (FDA) under the Human Cells, Tissues, and Cellular and Tissue-Based Products (HCT/Ps) framework. Products that are minimally manipulated and intended for homologous use may fall under Section 361, which involves a less burdensome regulatory pathway. However, most culture-expanded MSC products, including those intended for intervertebral disc regeneration, are likely to be considered more than minimally manipulated and/or used for non-homologous purposes. These products are therefore generally regulated under Section 351 as biological products, requiring an Investigational New Drug application, biologics licensing, and extensive preclinical and clinical evaluation [93,94,95].

Japan has adopted a distinct regulatory approach designed to accelerate the clinical translation of regenerative medicine products. Under this framework, regenerative therapies may receive conditional and time-limited approval after early evidence of safety and probable efficacy, with additional confirmation required through post-marketing surveillance. This model may facilitate earlier patient access and reduce barriers to clinical introduction; however, it also places greater emphasis on long-term follow-up and real-world evidence to confirm durability, efficacy, and safety [96].

These regional differences in terminology, product classification, approval pathways, and evidence requirements create practical barriers for MSC-based therapies. A product classified as an ATMP in the European Union may be regulated as a biological product in the United States and as a regenerative medical product in Japan [95,96,97]. Such differences complicate the harmonisation of manufacturing protocols, potency assays, release criteria, clinical endpoints, and post-marketing surveillance requirements. For intradiscal MSC therapy, these regulatory inconsistencies are particularly relevant because variability in cell source, culture expansion, dose, delivery method, and intended mechanism of action may directly influence regulatory classification. Greater international harmonisation of regulatory standards, together with transparent reporting of manufacturing procedures, potency testing, and clinical outcomes, will therefore be essential for the successful global translation of MSC-based therapies for intervertebral disc regeneration [96,97].

## 8. Biomaterials, Bioreactors, and Translational Engineering Strategies for Mesenchymal Stem Cell Therapy in Intervertebral Disc Regeneration

**Figure 4:**
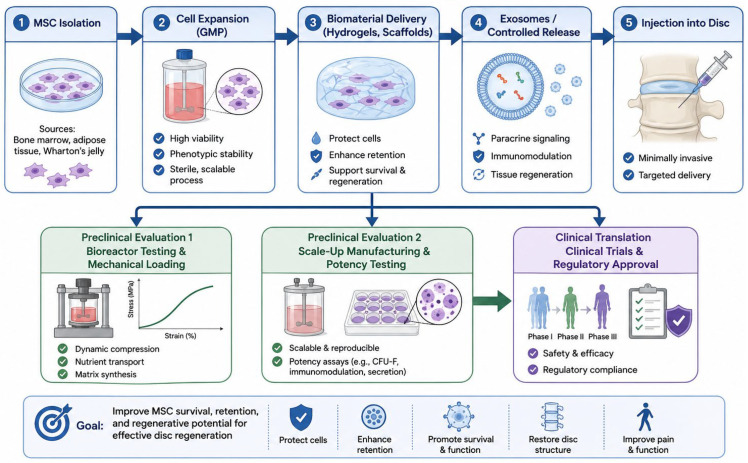
Schematic overview of bioengineering strategies to enhance MSC therapy for intervertebral disc regeneration. The translational pathway includes MSC isolation, in vitro expansion under GMP conditions, and delivery using biomaterial-based systems such as hydrogels and scaffolds, as well as exosome-based controlled release approaches, followed by intradiscal injection. Bioengineering tools, including dynamic bioreactors and mechanical loading systems, are employed during preclinical development to optimise cell function, survival, and regenerative potential. Subsequent large-scale manufacturing, potency testing, and regulatory approval are required for successful clinical translation. Overall, these strategies aim to improve MSC survival, retention, and therapeutic efficacy within the hostile intervertebral disc microenvironment.

Mesenchymal stem cell therapy for intervertebral disc regeneration has shown encouraging results in preclinical studies; however, poor cell survival, limited cell retention, and the harsh microenvironment of the degenerated disc remain major barriers to successful clinical translation. The intervertebral disc is characterised by hypoxia, low nutrient availability, acidic pH, and high mechanical loading, all of which negatively affect the viability and regenerative capacity of transplanted cells. To address these limitations, bioengineering strategies, including biomaterial-based delivery systems, bioreactor technologies, exosome-based therapies, and standardised manufacturing approaches, are increasingly being developed to enhance the therapeutic potential of MSC-based treatments (Figure 4) [88,89,90,91,92,98,99].

### 8.1. Biomaterials for Mesenchymal Stem Cell Delivery

Biomaterials play a critical role in improving MSC survival, retention, and differentiation within the degenerated intervertebral disc. Hydrogels are among the most widely investigated biomaterials for intradiscal MSC delivery due to their high water content, biocompatibility, and ability to mimic the native ECM [98,99]. Natural hydrogels such as alginate, collagen, hyaluronic acid, and fibrin have demonstrated the ability to support MSC viability and promote differentiation toward nucleus pulposus-like cells. Alginate hydrogels provide a three-dimensional microenvironment that supports proteoglycan synthesis, while collagen-based hydrogels promote cell adhesion and extracellular matrix production. Hyaluronic acid hydrogels are particularly attractive due to their similarity to native disc ECM components and their anti-inflammatory properties, whereas fibrin gels provide a biodegradable scaffold that supports cell attachment and tissue integration [98,99,100,101].

Injectable scaffolds represent another important strategy for minimally invasive MSC delivery. These biomaterials can be injected in liquid form and subsequently solidify in situ, allowing them to fill irregular disc spaces, improve cell retention, and reduce leakage from the injection site. Microsphere-based systems are also being investigated for controlled cell and growth factor delivery. These systems can protect MSCs from mechanical stress and provide sustained release of bioactive molecules that promote tissue regeneration and modulate inflammation [102].

Advanced fabrication techniques such as 3D bioprinting and nanofibrous scaffolds are being explored to better replicate the complex structure of the intervertebral disc. Nanofibrous scaffolds mimic the fibrous architecture of the annulus fibrosus and provide mechanical support while promoting cell attachment, alignment, and extracellular matrix deposition. 3D bioprinting allows for precise spatial distribution of cells and biomaterials, enabling the fabrication of constructs that more closely resemble native disc tissue architecture and mechanical behaviour [103,104]. Smart biomaterials represent an emerging area of research in disc regeneration. These include pH-responsive and oxygen-releasing biomaterials designed to address the hypoxic and acidic microenvironment of the degenerated disc. Oxygen-releasing scaffolds can improve cell survival by enhancing local oxygen availability, while pH-responsive materials can enable controlled release of therapeutic agents in response to the acidic conditions characteristic of disc degeneration [105].

The mechanical properties of biomaterials are critically important for successful disc regeneration. Scaffolds must withstand compressive loading while maintaining sufficient elasticity and viscoelastic behaviour to support cell survival and matrix production. Biomaterial stiffness has been shown to influence MSC differentiation, with softer matrices promoting nucleus pulposus-like phenotypes and stiffer matrices promoting fibrotic differentiation. In addition to mechanical strength, biomaterials must allow for adequate nutrient diffusion and waste removal, as the disc is an avascular tissue that relies on passive transport through the cartilaginous endplates. Scaffold porosity, permeability, and degradation rate therefore play a crucial role in maintaining cell viability and metabolic activity. Consequently, biomaterial-cell interactions, including mechanotransduction pathways, are key factors in determining regenerative outcomes [106].

### 8.2. Material-Assisted Delivery and Advanced Biofabrication Strategies

Material-assisted delivery systems have become a crucial element in enhancing the therapeutic efficacy of MSC-based strategies for intervertebral disc regeneration. Beyond simple cell injection, biomaterials provide structural support, improve cell retention, and protect transplanted cells from the hostile disc microenvironment. Hydrogels, injectable scaffolds, and microsphere-based systems not only reduce cell leakage but also enable controlled release of bioactive factors and extracellular vesicles, thereby prolonging regenerative signalling [106]. Recent advances in material-assisted regenerative strategies have increasingly focused on the development of smart, adaptive biomaterials that address both biological and biomechanical limitations of intervertebral disc regeneration. In particular, biofabrication technologies such as three-dimensional (3D) bioprinting and four-dimensional (4D) printing have significantly expanded the potential of biomaterial-assisted therapies [102,106,107].

Three-dimensional (3D) bioprinting of hydrogel-based scaffolds represents a versatile and rapidly evolving approach for intervertebral disc regeneration. Hydrogels function as multifunctional platforms whose physical and chemical properties, including pore size, microstructure, degradability, and stimulus-responsive behaviour, can be precisely engineered to support cell viability, differentiation, and extracellular matrix production. This tenability allows hydrogels to closely mimic the native extracellular matrix of the disc while serving as carriers for cells and bioactive molecules. Advanced biofabrication techniques enable the precise deposition of cell-laden biomaterials, facilitating the generation of complex structures that replicate key anatomical features of the intervertebral disc, including the nucleus pulposus and annulus fibrosus compartments [102,103]. Recent studies have demonstrated that such hydrogel-based constructs can support MSC survival, promote proteoglycan and collagen type II synthesis, and enhance disc-like tissue formation in preclinical models. Despite being in relatively early stages of development, 3D bioprinting is considered an indispensable manufacturing technology with significant potential for generating biomimetic, transplantable tissues for disc regeneration [102].

In parallel, four-dimensional (4D) printing approaches introduce time-dependent shape transformation and responsiveness to external stimuli, enabling the development of dynamic and adaptive biomaterials. A notable example is the development of near-infrared (NIR)-responsive, deployable, and self-fitting scaffolds fabricated from programmable polymer composites. These scaffolds can be implanted in a compact form using minimally invasive techniques and subsequently undergo rapid and tuneable shape recovery under NIR stimulation, allowing for precise adaptation to irregular defect geometries [107]. In preclinical studies, such systems have demonstrated excellent shape-memory properties, enhanced biological activity, and near-complete tissue regeneration in vivo, including increased alkaline phosphatase activity and mineral deposition. Although these technologies have primarily been investigated in bone tissue engineering, their underlying principles (minimally invasive delivery, improved mechanical conformity, and enhanced structural integration) are highly relevant to intervertebral disc regeneration, where similar challenges persist [99,102,103,107].

Despite these promising technological advances, translation into consistent and clinically meaningful outcomes remains limited. Biomaterial-assisted strategies, including hydrogels, scaffolds, and bioprinted constructs, have been shown to improve short-term cell retention, enhance MSC survival, and stimulate local regenerative signalling. However, their impact on long-term structural regeneration remains unclear. In particular, consistent restoration of disc height, biomechanical function, and durable extracellular matrix integrity has not yet been reliably demonstrated in clinical settings. Several factors contribute to this translational gap. Many biomaterial-based systems are evaluated in small animal models characterised by lower mechanical loading and more favourable metabolic conditions, which may overestimate therapeutic efficacy compared with the human spine. In addition, limitations such as restricted nutrient diffusion within larger constructs, mismatch between scaffold mechanical properties and native tissue, and insufficient long-term integration within the avascular and hostile disc microenvironment continue to hinder clinical success. Furthermore, there is a lack of direct comparative studies demonstrating clear superiority of material-assisted delivery over conventional intradiscal cell injection, particularly with respect to long-term outcomes [98,99,100,101,107].

Taken together, these findings suggest that while material-assisted strategies are a critical component of next-generation regenerative therapies, their current role remains primarily supportive rather than curative. Future research should focus on the development of multifunctional biomaterials capable of simultaneously enhancing cell survival, modulating the disc microenvironment, and providing long-term mechanical stability. The integration of responsive biomaterials, controlled-release systems, and advanced biofabrication technologies will likely be essential to achieve durable and clinically meaningful regeneration of the intervertebral disc [102,106,107].

### 8.3. Bioreactors and Mechanical Loading Systems

Bioreactor systems are increasingly used to study MSC behaviour under physiologically relevant conditions. Dynamic compression bioreactors simulate the mechanical loading experienced by the intervertebral disc during daily activities, allowing researchers to investigate how mechanical stress influences MSC viability, differentiation, and extracellular matrix production. These systems also allow for precise control of oxygen concentration, nutrient supply, pH, and mechanical loading, enabling the study of cell behaviour under conditions that closely mimic the native disc microenvironment. Disc organ culture systems represent another important experimental platform, as they preserve native disc structure and enable the evaluation of regenerative therapies in a controlled environment [108]. Mechanobiology plays a crucial role in MSC-based disc regeneration. Mechanical loading has been shown to regulate MSC differentiation, matrix synthesis, and cytokine production. Moderate dynamic compression promotes chondrogenic and nucleus pulposus-like differentiation, whereas excessive mechanical stress can reduce cell viability and inhibit regenerative processes. Understanding the relationship between mechanical loading and MSC behaviour is therefore essential for the development of effective regenerative therapies [109].

### 8.4. Exosome-Based Therapy

MSC-derived exosomes have emerged as a promising cell-free alternative to stem cell therapy. Exosomes are extracellular vesicles that contain proteins, lipids, and nucleic acids capable of modulating inflammation, promoting extracellular matrix synthesis, and inhibiting apoptosis in degenerated disc cells. Exosome-based therapies offer several advantages over cell-based therapies, including lower immunogenicity, improved safety profile, easier storage, and the potential for large-scale standardised manufacturing [110]. Exosomes can also be incorporated into biomaterial-based drug delivery systems for controlled release. Hydrogel-based delivery systems, microspheres, and nanoparticle carriers are being developed to provide sustained release of exosomes within the intervertebral disc, thereby prolonging their therapeutic effects and improving regenerative outcomes [111].

### 8.5. Manufacturing, GMP, and Standardisation and Regulatory Pathway for MSC-Based Therapies

One of the major barriers to clinical translation of MSC therapy is the lack of standardised manufacturing protocols. Large-scale cell expansion requires Good Manufacturing Practice (GMP)-compliant facilities, closed bioreactor systems, and strict quality control procedures. Closed-system bioreactors reduce contamination risk and enable reproducible large-scale MSC production. Potency assays are also essential to ensure consistent therapeutic efficacy, although defining reliable potency markers remains challenging due to the complex mechanism of action of MSCs, which involves paracrine signalling, immunomodulation, and extracellular vesicle secretion [112].

Standardisation of cell isolation, expansion, cryopreservation, and delivery protocols is necessary to ensure reproducibility across clinical studies. Scale-up production strategies, including automated bioreactor expansion systems, are being developed to enable widespread clinical use of MSC-based therapies and to reduce manufacturing costs while maintaining product consistency [109].

**Figure 5:**
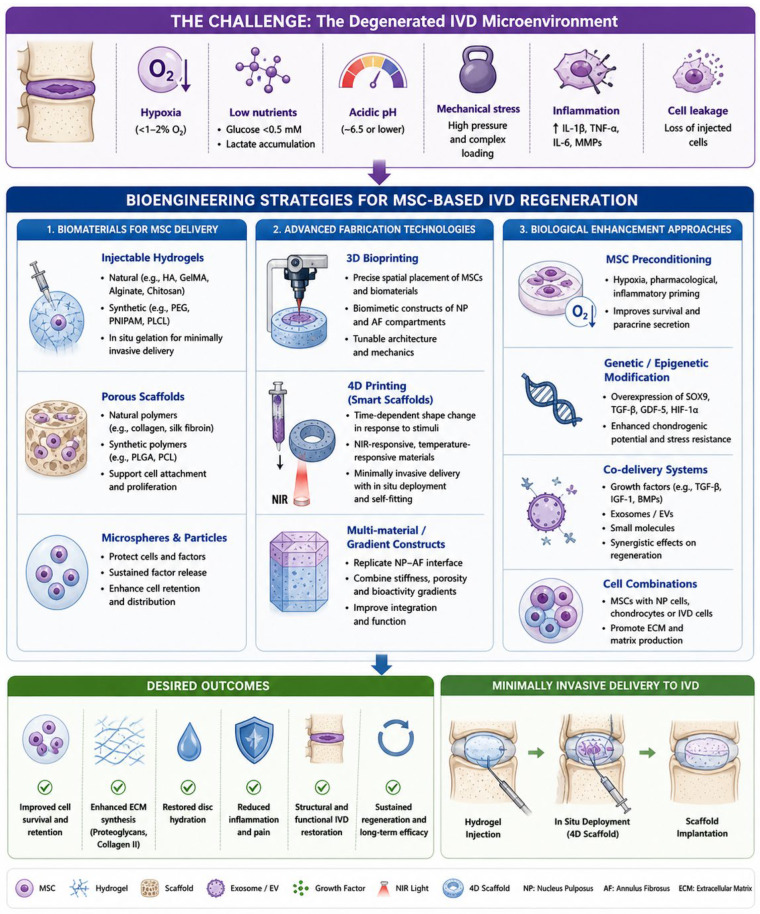
Key biomaterials and bioengineering strategies for MSC-based intervertebral disc regeneration. The figure summarises the major challenges of the degenerated intervertebral disc microenvironment and the corresponding bioengineering strategies designed to improve MSC-based therapies. The left panel illustrates the hostile intervertebral disc microenvironment, characterised by hypoxia (typically <1–2% O_2_), low nutrient availability (glucose < 0.5 mM), acidic pH (≈6.5 or lower), inflammation (elevated IL-1β, TNF-α, IL-6, and matrix metalloproteinases), mechanical stress, and cell leakage following intradiscal injection. These factors collectively impair MSC survival, retention, and regenerative function. The central panels present bioengineering strategies grouped into three main categories. (1) Biomaterials for MSC delivery include injectable hydrogels, porous scaffolds, microspheres, and functionalised matrices that enhance cell retention, protect against mechanical stress, and provide ECM-mimetic cues. (2) Advanced fabrication technologies, such as 3D bioprinting and 4D printing, enable the creation of biomimetic constructs with spatially controlled architecture, tuneable mechanical properties, and stimulus-responsive behaviour, including near-infrared (NIR)-triggered deployment. Multi-material and gradient constructs further replicate the native nucleus pulposus–annulus fibrosus interface. (3) Biological enhancement approaches include MSC preconditioning, genetic or epigenetic modification, co-delivery systems incorporating growth factors or extracellular vesicles, and cell combination strategies to improve regenerative potential and resilience to the disc microenvironment. The right panel highlights minimally invasive delivery strategies, including hydrogel injection, scaffold implantation, and in situ deployment of smart biomaterials, aiming to balance therapeutic efficacy with clinical feasibility. Cross-cutting design principles include biocompatibility, mechanical compatibility, controlled degradation, bioactivity, and scalability for clinical translation. Collectively, these approaches aim to improve MSC survival and retention, enhance extracellular matrix synthesis, restore disc hydration and biomechanics, reduce inflammation and pain, and ultimately achieve functional regeneration of the intervertebral disc.

The translational pathway for MSC-based therapies involves multiple stages, including cell sourcing, expansion, biomaterial integration, preclinical testing, clinical trials, and regulatory approval. Regulatory agencies classify MSC-based products as advanced therapy medicinal products, requiring strict quality control, safety testing, and long-term follow-up studies. Harmonisation of regulatory guidelines, manufacturing standards, and clinical outcome measures will be essential for the successful clinical translation of MSC-based therapies for intervertebral disc regeneration [110,111,112]. Key biomaterials and bioengineering strategies for mesenchymal MSC-based intervertebral disc regeneration are summarised in Figure 5.

## 9. Future Directions and Emerging Strategies

The development of regenerative therapies for degenerative disc disease is progressing rapidly, and MSC-based strategies remain at the forefront of these efforts. However, the successful translation of MSC therapy into routine clinical practice will require coordinated advances across multiple scientific and clinical domains. Future research should focus on improving cell survival, enhancing regenerative signalling, optimising delivery systems, and identifying patient populations most likely to benefit from biological therapies [113,114,115,116].

One promising approach involves improving the resilience and functionality of transplanted MSCs through cell preconditioning strategies. Exposure of MSCs to hypoxic conditions, inflammatory stimuli, or metabolic stress prior to implantation has been shown to enhance their survival and regenerative activity within the harsh intervertebral disc environment [2,3,4,113]. Hypoxic preconditioning, in particular, appears to activate adaptive metabolic pathways that allow MSCs to better tolerate the low-oxygen conditions present within the nucleus pulposus. Experimental studies have demonstrated that preconditioned MSCs exhibit increased secretion of trophic factors, improved extracellular matrix production, and reduced apoptosis following transplantation [114].

Another emerging strategy involves genetically engineered MSCs designed to enhance regenerative potential. Advances in gene editing technologies, including CRISPR-based systems, enable targeted modification in MSCs to increase the expression of anabolic growth factors such as growth differentiation factor-6 (GDF-6), transforming growth factor-β (TGF-β), and bone morphogenetic proteins. These engineered cells may promote more efficient differentiation toward a nucleus pulposus-like phenotype and stimulate extracellular matrix regeneration within the degenerated disc [117,118].

Increasing attention is also being directed toward cell-free regenerative therapies, particularly those based on extracellular vesicles and exosomes derived from MSCs. These nanosized vesicles contain proteins, lipids, and regulatory RNAs that can modulate gene expression and cellular behaviour in recipient cells. MSC-derived exosomes have demonstrated anti-inflammatory, anti-apoptotic, and matrix-promoting effects in experimental models of disc degeneration [113,119]. Because they do not contain living cells, exosome-based therapies may circumvent several safety concerns associated with stem cell transplantation, including immune rejection, uncontrolled differentiation, and tumour formation [113,119].

Another important research direction involves the integration of tissue engineering approaches with stem cell therapy [114,119]. Biomaterial scaffolds, hydrogels, and injectable matrices are being developed to improve the retention and viability of implanted cells within the pressurised disc environment. These biomaterials can provide mechanical support while also serving as carriers for growth factors or extracellular vesicles, enabling controlled release of regenerative signals over time. Advances in three-dimensional bioprinting may further enable the fabrication of disc-like constructs that replicate the structural organisation of the native intervertebral disc [120,121,122,123].

Improved patient stratification and precision medicine approaches may also play an important role in the future development of regenerative therapies. Degenerative disc disease is a heterogeneous condition with diverse underlying mechanisms [16,113,121]. Molecular biomarkers, advanced imaging techniques, and machine-learning-based analysis may help identify patient subgroups most likely to respond to MSC-based therapies. Early-stage disc degeneration, in particular, may represent a more suitable therapeutic target than advanced structural collapse, where regenerative potential may already be severely limited [122,123,124].

Finally, future clinical progress will depend on the implementation of large-scale, multicentre randomised controlled trials with long-term follow-up. These studies will be essential to determine the durability of therapeutic effects, evaluate safety profiles, and establish optimal treatment protocols. Standardisation of MSC sourcing, expansion, delivery, and quality control procedures will also be necessary to ensure reproducibility and regulatory approval [2,3,4,121,122].

In summary, the next generation of regenerative therapies for intervertebral disc degeneration will likely involve a combination of stem cell biology, biomaterial engineering, gene therapy, and precision medicine approaches. By integrating advances in these fields, it may become possible to develop safe and effective biological therapies capable of restoring disc structure, reducing chronic pain, and improving quality of life for patients with degenerative spine disorders [2,23,24,121,122].

## 10. Conclusions

Mesenchymal stem cell therapy represents a promising regenerative approach for the treatment of intervertebral disc degeneration, with preclinical studies and early clinical trials demonstrating encouraging effects on pain relief, functional improvement, and, in some cases, disc structure. However, despite these promising findings, consistent and predictable clinical outcomes have not yet been achieved. The limited clinical translation of MSC-based therapies is primarily associated with poor cell survival within the harsh intervertebral disc microenvironment, insufficient cell retention following implantation, variability in cell sources and manufacturing protocols, and a lack of standardised delivery methods and outcome measures. In addition, regulatory and manufacturing challenges further complicate the development of reproducible and scalable MSC-based therapies.

Future progress in this field will depend on improving cell survival and retention, developing biomaterial-supported delivery systems, standardising cell manufacturing and quality control procedures, and implementing large-scale, multicentre clinical trials with long-term follow-up and objective outcome measures. Emerging strategies, including hypoxic preconditioning, gene-modified MSCs, and cell-free therapies based on extracellular vesicles and exosomes, may help overcome some of the current biological and technical limitations.

Ultimately, the successful translation of MSC therapy for intervertebral disc degeneration will require coordinated advances in stem cell biology, biomaterial engineering, biomechanics, and clinical trial design. With improved standardisation, better preclinical models, and rigorous clinical evaluation, MSC-based therapies may in the future become a viable biological treatment option capable of addressing the underlying mechanisms of disc degeneration rather than only alleviating symptoms.

## Figures and Tables

**Table 1:** Representative preclinical animal studies investigating MSC-based therapy for intervertebral disc degeneration. NR, not reported.

Study	Model	Cell Source	Cell Dose	Delivery Method	Primary Outcome Measure	Key Findings	Limitations
Nishimura & Mochida (1998) [36]	Rat disc degeneration model	Autologous nucleus pulposus cells	NR	Intradiscal injection	Histology, disc structure	Reimplantation delayed degeneration of the annulus fibrosus and preserved nucleus pulposus tissue compared with untreated controls	Small animal model; acute injury model; limited translational relevance
Crevensten et al. (2003) [38]	Rat	Bone marrow-derived MSCs	NR	Intradiscal injection	Disc height, cellularity	Increased disc height and cellularity; evidence of extracellular matrix regeneration	Short follow-up period; biomechanical differences from human spine
Sakai et al. (2005) [39]	Rabbit	MSCs	NR	Intradiscal injection	Proteoglycan, collagen II	Differentiation into nucleus pulposus-like cells; increased proteoglycan and type II collagen production; improved disc hydration	Animal model with lower mechanical loading than humans
Clarke et al. (2014) [37]	In vitro differentiation model	Bone marrow-derived MSCs stimulated with GDF-6	NR	Growth factor–induced differentiation	Gene expression	Increased expression of nucleus pulposus–specific genes and discogenic phenotype markers	In vitro model; no in vivo biomechanical environment
Steffen et al. (2017) [43]	Canine model	MSCs	NR	Intradiscal injection	Mobility, pain	Improved mobility and reduced pain behaviour despite minimal radiological changes	Limited structural change

**Table 2:** Clinical studies evaluating MSC therapy for degenerative disc disease. Across clinical studies, MSC therapy consistently demonstrates improvement in pain and functional outcomes; however, radiological evidence of structural disc regeneration remains inconsistent, suggesting that clinical improvement may be mediated primarily through anti-inflammatory and paracrine mechanisms rather than true tissue regeneration. Where reported, cell doses ranged from ~10^6^ to 10^7^ MSCs per disc, although reporting was inconsistent across studies. NR, not reported.

Study	Study Design	Patients (n)	Cell Source	Number of Cells Injected	Delivery Method	Primary Outcome Measure	Follow-Up Period	Main Outcomes	Limitations
Yoshikawa et al. (2010) [47]	Case report	2	Autologous BM-MSCs	NR	Intradiscal injection	Pain, MRI signal	NR	Reduced pain and improved T2-weighted MRI signal intensity, suggesting improved disc hydration	Extremely small sample size; no control group
Orozco et al. (2011) [48]	Prospective clinical study	10	Autologous MSCs	~10^6^–10^7^	Intradiscal injection	Pain, function	12 months	Significant pain reduction; improved functional capacity; increased disc hydration but no significant increase in disc height	No randomisation; small cohort
Pettine et al. (2015) [49]	Prospective cohort	26	Autologous BM-MSCs	~10^6^–10^7^	Intradiscal injection	Pain, disability	24 months	Significant reduction in pain scores and disability indices; improved functional outcomes	No placebo control; heterogeneous patient population
Centeno et al. (2017) [50]	Observational study	33	Autologous MSCs	NR	Intradiscal injection	Pain, MRI	NR	Improved clinical symptoms and disc morphology; reduction in disc protrusion on imaging	Observational design; potential selection bias
Elabd et al. (2016) [51]	Pilot study	5	Hypoxia-preconditioned BM-MSCs	NR	Intradiscal injection	Pain, mobility	NR	Clinical improvement, increased mobility, and reduced pain	Very small sample size; short follow-up
Noriega et al. (2017) [52]	Controlled clinical trial	NR	MSCs	NR	Intradiscal injection	VAS, MRI	12 months	Improved VAS pain scores and MRI parameters compared with control group	Small sample size; limited statistical power
Amirdelfan et al. (2021) [53]	Randomised controlled trial	100	MSCs	~10^6^–10^7^	Intradiscal injection	Pain, function	24 months	Significant reduction in pain and improved functional outcomes; favourable safety profile	Variability in cell preparation and delivery; long-term efficacy uncertain

**Table 3:** Comparison of preclinical vs. clinical outcomes, emphasising the translational gap.

Outcome	Animal Studies	Clinical Studies
Disc height	Improved	Rarely improved
ECM production	Increased	Unclear
Disc hydration	Improved	Sometimes improved
Pain	Not primary outcome	Improved
Function	Improved	Improved
Structural regeneration	Yes	Inconsistent
Cell survival	Higher	Low
Mechanical load	Low	High

**Table 4:** Key barriers to clinical translation of MSC therapy for intervertebral disc degeneration. These barriers illustrate that the main limitation of MSC therapy for intervertebral disc degeneration is not the lack of biological potential, but rather the combination of biomechanical, microenvironmental, technical, and regulatory challenges that limit consistent clinical translation.

Category	Barrier	Description	Impact on Clinical Translation
Biomechanical	High mechanical loading	Human intervertebral discs are exposed to continuous compression, torsion, and shear forces	Reduces survival and retention of implanted cells
Microenvironment	Hypoxia, acidity, low nutrient supply	Degenerated discs have limited glucose and oxygen and increased lactic acid	Impairs MSC metabolism and regenerative capacity
Biological	Limited cell survival	Implanted MSCs may survive only a few weeks after injection	Limits long-term regenerative effect
Mechanistic	Unclear mechanism of action	MSCs may act via paracrine signalling rather than differentiation	Makes optimisation of therapy difficult
Technical	Cell leakage after injection	Cells may escape through annular fissures or needle tract	Reduces effective therapeutic dose
Manufacturing	Variability in MSC source and expansion	Differences in donor age, tissue source, and culture conditions	Leads to inconsistent clinical outcomes
Clinical	Outcome measurement limitations	Pain relief does not necessarily correlate with disc regeneration	Makes interpretation of clinical success difficult
Regulatory	Complex approval pathways	Different regulatory frameworks across regions	Slows clinical translation
Economic	High production cost	GMP manufacturing and quality control are expensive	Limits widespread clinical use

## Data Availability

The data supporting the findings of this study are available from the corresponding author upon reasonable request.
